# Should whole body vibration be used for falls prevention in older people living in the community?

**DOI:** 10.1186/s13643-025-02946-5

**Published:** 2025-10-31

**Authors:** Suzanne M. Dyer, Wing S. Kwok, Rik Dawson, Ian D. Cameron, Catherine Sherrington

**Affiliations:** 1 Flinders University, College of Medicine and Public Health, Flinders Health and Medical Research Institute, GPO Box 2100, Bedford Park, Adelaide, SA Australia; 2 School of Public Health, Faculty of Medicine and Health, Institute for Musculoskeletal Health, The University of Sydney, Sydney Local Health District, Sydney, NSW Australia; 3 John Walsh Centre for Rehabilitation Research, Northern Sydney Local Health District and the University of Sydney, St. Leonards, NSW Australia

**Keywords:** Older people, Community, Falls, Care homes, Whole body vibration

## Abstract

The Canadian Task Force on Preventive Health Care has recently published a systematic review and network meta-analysis that concludes that whole-body vibration (WBV) has moderate-certainty evidence for falls prevention for older people living in the community. However, as Cochrane Collaboration falls prevention review authors and clinicians, we suggest that when the range of possible effects captured with the 95% confidence intervals, the likelihood of adverse events and the lack of evidence for effectiveness in older people living in care facilities are taken into account, that this intervention should be implemented with caution in this population. Outside of the clinical trial setting, WBV in this population should only be implemented following an individually tailored assessment and with guidance from an appropriately trained health professional.

## Background

The Canadian Task Force on Preventive Health Care published a systematic review and network meta-analysis in 2024 conducted to inform recommendations in an in-progress guideline on falls prevention for older people living in the community [1]. Providing updated guidance on this important public health issue is to be commended and the inclusion of reviews of patient preferences is an important improvement in guideline formulation methodology. The key conclusions for long-duration balance/resistance and group Tai Chi exercise interventions and home hazard modification to prevent falls concur with the findings of the most recent Cochrane Collaboration reviews, meta-analyses and the World Falls Guidelines [2–6]. However, as authors of Cochrane Collaboration systematic reviews of all interventions to prevent falls in care facilities [7, 8], and exercise to prevent falls in older people living in the community [2], we suggest that the conclusion on the benefit of whole-body vibration (WBV), based on moderate-certainty evidence for reducing fallers, should be implemented with caution [1].

### Network meta-analysis evidence for WBV in the community

The evidence informing the recommendation for WBV in the published network meta-analysis comprises two randomised controlled trials (RCTs) [9, 10] of 758 participants, with the larger trial (710 participants) conducted in post-menopausal women [9]. In the smaller trial, training was conducted three times a week for 8 weeks, with falls outcomes measured at 12 months [10]. Training effects for exercise interventions are not maintained after the intervention is discontinued [3]. As researchers (all authors), practicing physiotherapists (CS, RD, WK) and a professor of rehabilitation medicine and clinician working in rehabilitation medicine with older people (IDC), we expect that any effect of WBV is also unlikely to have been maintained at 12-month follow-up [3]. Thus, the majority of the evidence comes from the larger trial of over 700 participants, delivering supervised WBV for 20 min daily for 5 days per week over 18 months [9]. The review authors have indicated that delivery of this dose of the intervention may not be feasible or acceptable outside of these trials, a view with which we concur [1].

The 95% confidence interval (CI) for the effectiveness of WBV in reducing the number of fallers and fractures includes the possibility of an increase in these events (CINeMA moderate certainty evidence; fallers prevented per 100 treated = 12, 95% CI, − 1 to 22; people with fractures prevented per 100 = 3, 95% CI − 4 to 4), with low certainty evidence for reducing the number of falls (falls prevented per 100 people 54, 95% CI 0 to 82). Whilst this uncertainty is captured by downgrading the certainty of the evidence for imprecision by half of one step, with additional downgrading for heterogeneity between trial outcomes, our view is that the conclusion of moderate certainty evidence for benefit implies a greater level of confidence in the use of this intervention than is warranted. In addition, the review conducted to inform the outcome valuation found that fractures from falls may be more important than falls (based on the disutility values) and named WBV as an intervention with low certainty for benefit in reducing fractures. However, as the network meta-analysis finding for WBV was for a reduction of people experiencing fractures of 3 per 100, but with a 95% CI ranging from an increase to a decrease in fractures of 4 per 100, we suggest that listing WBV as supported by the outcome valuation does not appear to be consistent with the range of plausible effects, considering that the CIs include the possibility of an increase in fractures of 4 per 100. In addition, WBV is associated with moderate certainty evidence for 5 adverse events per 100 people, although not serious (any adverse event 4.7 per 100 people, 95% CI 2.8 to 7.3; serious adverse events = 0 per 100 people, 95% CI 0 to 0.9).

### Cochrane Collaboration systematic review evidence for WBV in care facilities

In a recently published updated Cochrane Collaboration review on the prevention of falls in older people living in care facilities [7, 8], we have found that the evidence overall for WBV is too sparse to inform a clear conclusion. The two included trials also delivered WBV at a relatively high dose of three times weekly for 6 months [11] and twice weekly for 12 months [12]. However, outcomes from the trials indicated either no effect or a non-statistically significant trend to an increase in falls (Table 1). Data on falls could not be obtained from two additional identified trials, despite an indication in the trials’ registry record [13, 14] or conference abstract [15] that falls data would be available. Overall, these data indicate a lack of evidence for the effectiveness of WBV in residents of care facilities. We suggest that this also indicates that the use of WBV in older people living in the community with frailty or an increased falls risk should also be undertaken with caution.

**Table 1:** Effectiveness of whole body vibration to reduce falls in randomised controlled trials in care facilities^a^

Study	Participants in analysis (*N*)	Falls measure	Results^b^
**At end of the intervention period**			
Buckinx et al. 2014 [11]	62	Rate ratio	1.34 (95% CI 0.73 to 2.45)
Buckinx et al. 2014 [11]	62	Risk ratio	1.00 (95% CI 0.46 to 2.19)
**After period of post-intervention follow-up**			
Buckinx et al. 2014 [11]^c^	62	Rate ratio	0.96 (95%CI 0.58 to 1.60)
Koike 2019 [12, 16]^d^	171	Rate ratio	1.51 (95%CI 1.15 to 1.98)
*Rate ratio pooled estimate of effect*			*1.27 (95%CI 0.83 to 1.95)*
Buckinx et al. 2014 [11]^c^	62	Risk ratio	0.88 (95%CI 0.54 to 1.43)
Koike 2019 [12, 16]^d^	171	Risk ratio	1.38 (95%CI 0.97 to 1.96)
*Risk ratio pooled estimate of effect*			*1.14, 95%CI 0.74 to 1.76*

^a^Data reproduced from Dyer et al. 2025 [3]

^b^Outcomes and meta-analysis determined using generic inverse variance method in Review Manager, as described in the associated Cochrane Collaboration reviews [7, 8]

^c^Outcomes at 12 months, 6 months post-intervention

^d^Outcomes at 18 months, 6 months post-intervention

## Conclusion

In summary, a conclusion of moderate certainty evidence for benefit of WBV to prevent falls and fractures in older people in the community indicates a greater level of confidence in the intervention than the evidence supports when taken as a whole. The range of possible effects captured within the 95%CIs, the increased likelihood of adverse events (even though not serious) and the lack of evidence for effectiveness in older people living in care facilities are important considerations. Our authorship team comprises a group of experts in evidence-based falls prevention (all authors) including professors of rehabilitation medicine (IDC) and physiotherapy (CS) as well as practicing physiotherapists in aged care (RD, WK) and the president of the Australian Physiotherapy Association (RD). Our expert and clinical opinion is that the use of WBV for older people in the community should only be implemented outside of the clinical trial setting with caution, following an individually tailored assessment and with guidance from an appropriately trained health professional.

## Data Availability

Data supporting the findings in this article are also included in the Cochrane Collaboration review “Interventions to prevent falls in older people living in care facilities” [8].

## Abbreviation

WBV: Whole-body vibration
